# Novel biomarkers to predict treatment response and prognosis in locally advanced rectal cancer undergoing neoadjuvant chemoradiotherapy

**DOI:** 10.1186/s12885-023-11354-8

**Published:** 2023-11-12

**Authors:** Bingjie Guan, Meifang Xu, Rong Zheng, Guoxian Guan, Benhua Xu

**Affiliations:** 1 Department of Radiation Oncology, Fujian Medical University Union Hospital, Fuzhou, China; 2 Department of Radiation Oncology, The First Affiliated Hospital of Fujian Medical University, Fuzhou, China; 3 Department of Pathology, Fujian Medical University Union Hospital, Fuzhou, China; 4 Fujian Key Laboratory of Intelligent Imaging and Precision Radiotherapy for Tumors, Fujian Medical University, Fuzhou, China; 5 Clinical Research Center for Radiology and Radiotherapy of Fujian Province (Digestive, Hematological and Breast Malignancies), Fuzhou, China; 6 Department of Colorectal Surgery, The First Affiliated Hospital of Fujian Medical University, Fuzhou, China

**Keywords:** Locally advanced rectal cancer, Neoadjuvant chemoradiotherapy, Treatment response, Prognosis, Immune infiltration

## Abstract

**Purpose:**

To identify genes associated with treatment response and prognosis for locally advanced rectal cancer (LARC) patients receiving neoadjuvant chemoradiotherapy (NCRT).

**Methods:**

In our cohort, gene expression profiles of 64 tumor biopsy samples before NCRT were examined and generated. Weighted gene co-expression network analysis was performed to identify gene modules. External validation datasets included GSE3493, GSE119409, and GSE133057. The expression of candidate genes was evaluated using immunohistochemistry (IHC). TIMER was used to assess immune infiltration.

**Results:**

We identified and validated the capability to predict the treatment response of CCT5 and ELF1 using our data and external validation datasets. The trends of survival differences of candidate genes in the GSE133057 dataset were similar to our cohort. High levels of CCT5 and ELF1 expression were associated with NCRT resistance and poor prognosis. Furthermore, the expression of CCT5 and ELF1 were also assessed in 117 LARC patients’ samples by the IHC method. Based on IHC results and Cox analysis, the risk score model with CCT5 and ELF1 was constructed and performed well. The risk score was an independent prognostic factor for progression-free survival and overall survival in LARC patients and was then used to build nomogram models. The underlying mechanisms of CCT5 and ELF1 were explored using gene set enrichment analysis. The underlying pathway including apoptosis, cell cycle, and other processes. CCT5 and ELF1 expressions were significantly correlated with immune cell infiltration.

**Conclusion:**

CCT5 and ELF1 were determined as biomarkers for treatment response and prognosis in LARC patients. The risk score model and nomograms helped predict treatment response and survival outcomes for LARC patients undergoing NCRT.

**Supplementary Information:**

The online version contains supplementary material available at 10.1186/s12885-023-11354-8.

## Introduction

Colorectal cancer remains a leading disease burden and ranks second in cancer-related mortality worldwide [1, 2]. Preoperative neoadjuvant chemoradiotherapy (NCRT) presents advantageous features in tumor downstaging, surgical resection rates, and anus conservation rates [3–5]. Currently, NCRT followed by radical surgery is a mainstay of therapeutic strategy for patients with locally advanced rectal cancer (LARC). However, roughly 15-45% of patients exhibit therapy resistance and are likely to suffer from potential complications and toxicity of NCRT, which must not be neglected [6]. Thus, identifying the regulator genes of NCRT is crucial to improving the treatment efficacy.

Previous studies have reported differentially expressed genes (DEGs) between NCRT-sensitive patients and NCRT-resistant patients and potential markers to predict tumor regression grading (TRG) [7–10]. Whereas, the prognostic value of the predictor was not explored in several studies. The neoadjuvant rectal (NAR) score has recently been proposed as a composite endpoint for LARC patients to predict clinical outcomes [11, 12]. A low NAR score indicates a positive treatment response and a better prognosis. In view of that, joint analysis of NAR score, TRG, and prognosis may contribute to a better understanding of NCRT regulatory factors and clinical outcomes.

In this study, we performed gene expression profiles on tumor biopsy samples from patients with LARC undergoing NCRT. Weighted gene co-expression network analysis (WGCNA) was used to determine NAR score-related modules and identify candidate genes with predictive and prognostic significance. Patient tissue samples and external datasets were used for validation.

## Method

### Patients and clinical data collection

A total of 64 patients with LARC undergoing NRCT between 2015 and 2018 in Fujian Medical University Union Hospital were enrolled. Specifically, as we previously reported [13], the radiotherapy consisted of a 45 Gy dose in 25 fractions over five weeks and a boost dose of 5.4 Gy for the tumor. And concurrent chemotherapy was as follows: oral capecitabine 825 mg/m^2^ twice per day for two weeks. Patients received radical surgery after 6 to 8 weeks from the last dose of radiotherapy. All patients were recommended to receive adjuvant chemotherapy after surgery. Tumor biopsy samples, used for gene expression profiles analysis, were obtained from colonoscopy before NCRT. In addition, LARC patients undergoing NRCT and radical surgery between 2012 and 2014 were also included. Their colonoscopy samples before neoadjuvant treatment were collected to validate and further identify the protein expression of candidate genes. This study was approved by the Institutional Review Board of Fujian Medical University Union Hospital (2019KY006).

TRG was used to assess pathological response to NCRT. In detail, TRG 0, no residual tumor cells; TRG 1, near-complete regression with tumor cells individually or in small groups; TRG2, residual tumor cells with a desmoplastic response; and TRG 3, minimal or no regression. Patients with TRG 0 and TRG 1 were classified as NCRT-sensitive groups, while TRG 2 and TRG 3 as NCRT-resistant groups. NAR score was calculated based on the equation: [5ypN–3 (cT–ypT) + 12]² /9.61 [12]. Hereinto, cT refers to clinical T stage (value: 1, 2, 3, 4), ypT refers to pathological T stage (value: 0, 1, 2, 3, 4), and ypN refers to pathological nodal status (value: 0, 1, 2).

### Gene expression analysis

The total RNA was extracted from tumor biopsy samples using Trizol reagent (Invitrogen) according to the manufacturer’s instructions. NanoDrop ND-1000 monitored RNA quality control and quantification. Labeling, hybridization, and scanning were carried out according to standard protocols. Then, data quality control and normalization were performed.

### DEGs identification and enrichment analysis

The DEGs were identified using the R “limma” package. Genes with |log fold change (logFC)|> 0.5 and p-value < 0.05 were determined as DEGs between the NCRT-sensitive and the NCRT-resistant group. Visualization and comparison of DEGs use volcano plots and heatmaps. Gene Ontology (GO) and Kyoto Encyclopedia of Genes and Genomes (KEGG) were used to explore the biological functions of DEGs by the R “clusterProfiler” package.

### WGCNA

WGCNA was carried out using the R “WGCNA” package [14]. That is, the soft threshold power was set, and the topological overlap matrix (TOM) was calculated. Modules were determined by the Dynamic Tree Cut method. Then, high similarity modules were merged by clustering analysis. The correlation between modules and clinicopathological features is calculated.

### Immunohistochemical analysis

The protein expression of the candidate genes was assessed by immunohistochemistry. Immunohistochemical staining for candidate genes was conducted as described earlier [15]. The following antibodies were used: anti-FBXO7 (203,049-T40, Sino Biological, China), anti-GSTT4 (bs-16345R, Bioss, China), anti-CCT5 (11603-1-AP, Proteintech, China), and anti-ELF1 (22565-1-AP, Proteintech, China), and anti-SLC44A1 antibody (14687-1-AP, Proteintech, China). Immunohistochemical results were scored using a semi-quantitative scoring method. Specifically, data were collected from random visual fields of five different areas. The intensity of staining was scored as 0 (negative), 1 (light yellow), 2 (brown), and 3 (deep brown). The percentage of positive cells was scored as 0 (< 5%), 1 (5–25%), 2 (25–50%), 3 (50–75%), and 4 (> 75%). The two values were multiplied and calculated as immunohistochemical scores. Scores of 0–4 were considered low expression, and those with scores above 4 were classified as high expression.

### External validation datasets

External validation datasets were obtained from Gene Expression Omnibus (GEO) database. The GSE3493, including 46 LARC patients undergoing NRCT, was identified to assess the gene expression of the candidate genes between NCRT-sensitive groups and NCRT-resistant groups. After excluding samples with unknown TRG information, a total of 56 LARC patients undergoing NRCT in GSE119409 were also included in the external validation. The GSE133057 (33 LARC patients) was adopted for the validation of survival outcomes.

### Gene set enrichment analysis

Gene set enrichment analysis (GSEA) was employed to investigate the potential biological pathways of candidate genes. LARC patients were enrolled in high and low-expression groups of candidate genes based on the median expression. The false discovery rate (FDR) < 0.25 and *P* < 0.05 were accepted as statistically significant.

### Immune infiltration analysis

The relationship between gene expression and immune cells infiltration (including B cell, CD8 + T cell, CD4 + T cell, macrophage, neutrophil, and dendritic cell) was explored using Tumor IMmune Estimation Resource (TIMER).

### Drug-sensitive analysis

The drug sensitivity dataset was obtained from the CellMiner website [16]. Pearson’s correlation test determined the correlation between target genes and drug sensitivity. These correlation results were visualized using the R package ggplot2.

### Statistical analysis

Statistical analysis was carried out with R software (version 4.1.2), GraphPad Prism 8, and SPSS (version 22). X-tile software was adopted to determine the cut-off points for the gene expression of the candidate genes. T-tests or non-parametric tests were done to test differences between continuous variables. The categorical data were analyzed by Fisher’s exact or Chi-square tests. Kaplan–Meier (KM) analysis was used to estimate survival outcomes using a log-rank test. Correlations were evaluated using Pearson correlation test. Receiver operating characteristics (ROC) analysis was performed, and the area under the curve (AUC) was calculated to assess the TRG and predictive survival capacities of the candidate genes. Cox proportional hazards regression analysis identified independent risk factors for progression-free survival (PFS) and overall survival (OS). A nomogram was created based on the above factors by the R “rms” package. The calibration curve was used to evaluate the performance of the model. *P*-value < 0.05 was considered statistically significant.

### Result

#### DEGs and functional enrichment

**Fig. 1:**
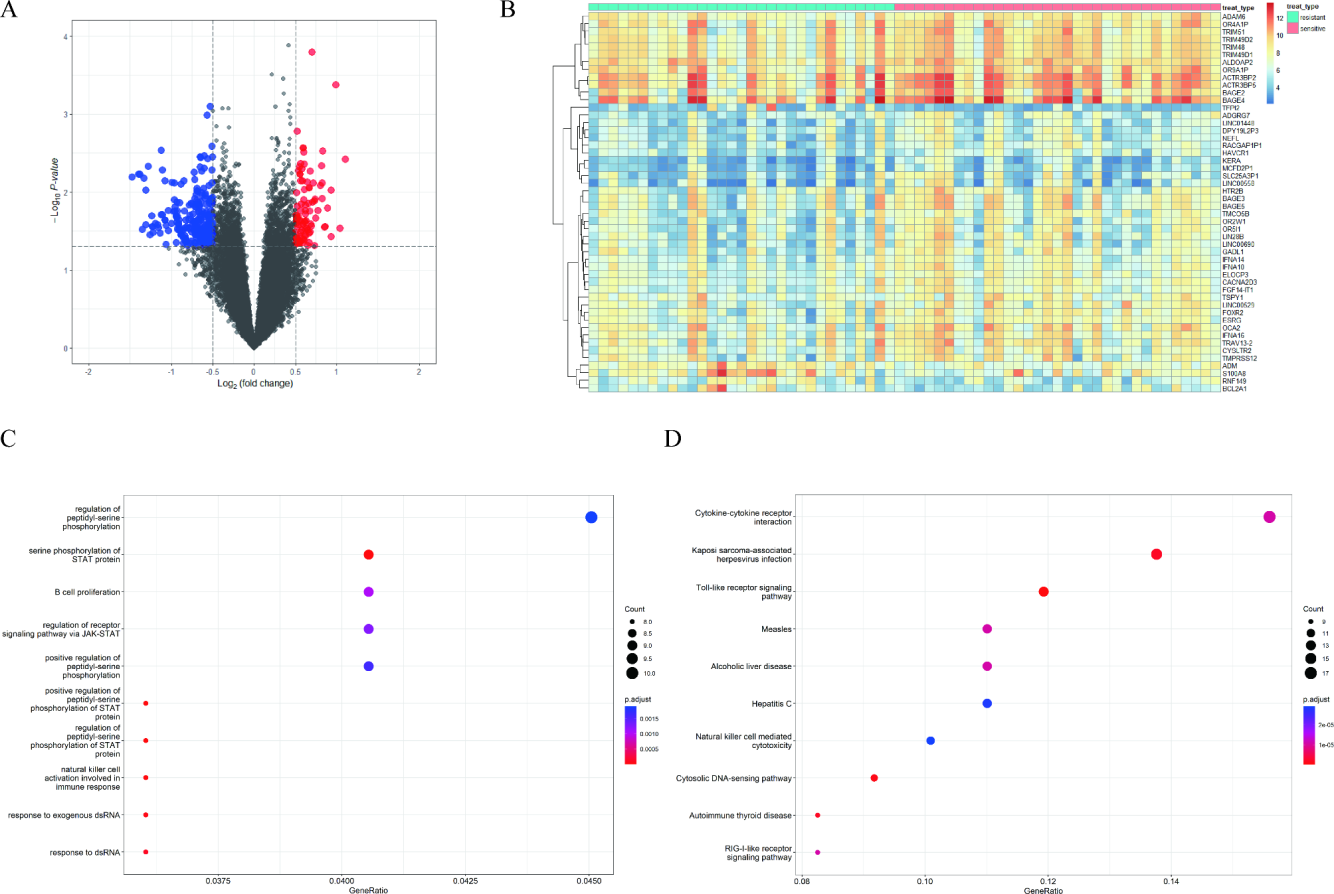
**DEGs between NCRT-sensitive patients and NCRT-resistant patients and functional enrichment** In 64 LARC patients, (**A**) Volcano plot of DEGs; (**B**) Heatmap of DEGs; (**C**) Top 10 pathways of GO enrichment analysis; (**D**) Top 10 pathways of KEGG functional enrichment LARC: locally advanced rectal cancer; DEGs: differentially expressed genes; NCRT: neoadjuvant chemoradiotherapy; GO: Gene Ontology; KEGG: Kyoto Encyclopedia of Genes and Genomes

The gene expression profile based on gene chips was obtained from 64 LARC patients (Supplementary Table 1) before NCRT in our cohort. A total of 333 DEGs were identified between NCRT-sensitive patients and NCRT-resistant patients. Among these, compared to the NCRT-sensitive group, 94 genes are upregulated, and 239 genes are downregulated in the NCRT-resistant group. Heatmap and volcano plot were shown in Fig. 1A and B. We performed GO and KEGG analyses to explore the potential biological significance of DEGs. GO analyses revealed that the DEGs were significantly enriched in serine phosphorylation of STAT protein, natural killer cell activation involved in immune response, response to exogenous dsRNA, and so forth (Fig. 1C). Furthermore, the top 10 enriched KEGG pathways are presented in Fig. 1D, including cytokine-cytokine receptor interaction, the Toll-like receptor signaling pathway, and cytosolic DNA-sensing pathway, respectively.

### Identification of candidate genes

**Fig. 2:**
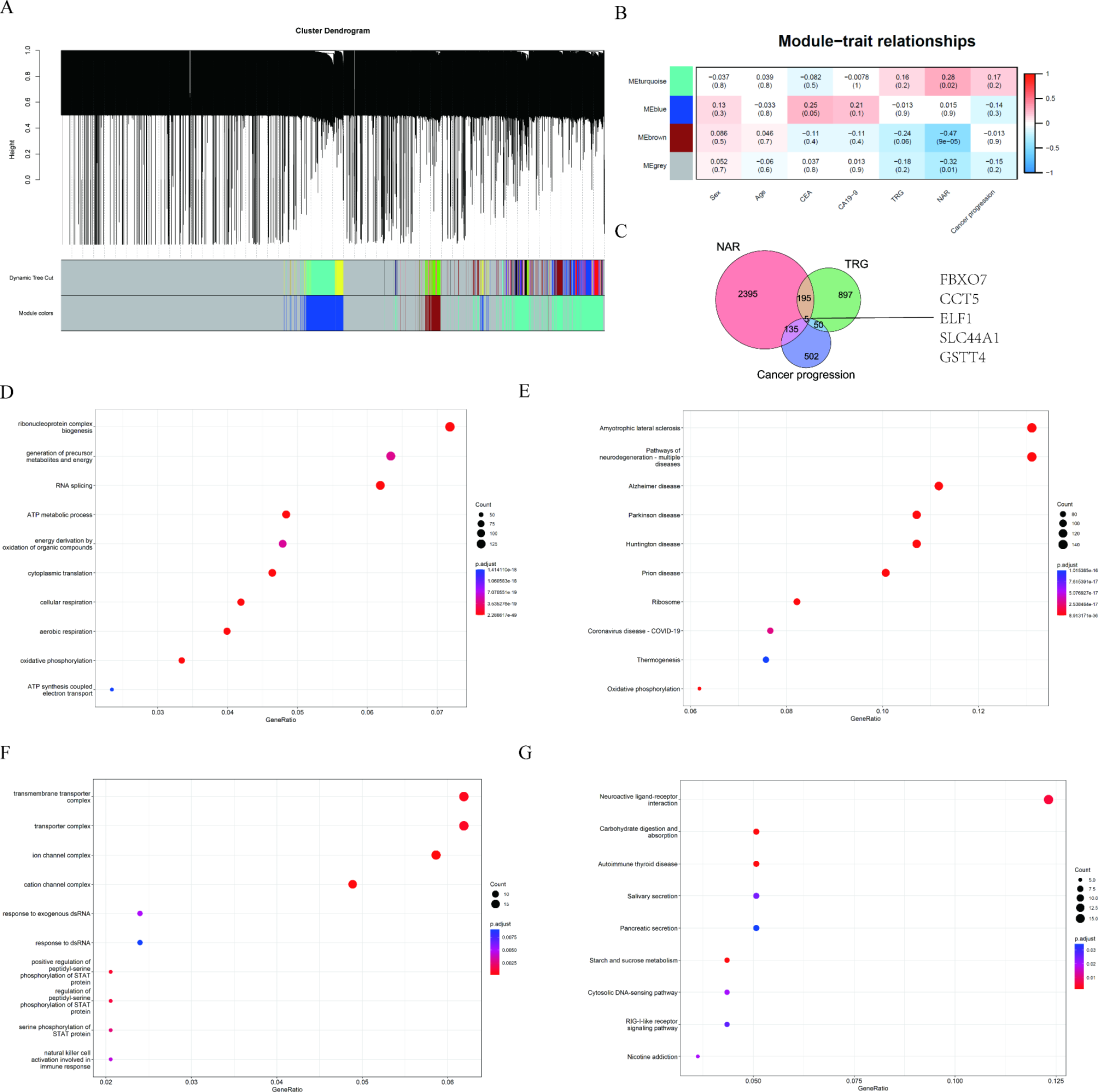
**WGCNA analysis** In 64 LARC patients, (**A**) Identification of WGCNA modules dynamic tree cut method; (**B**) The relationship between modules and clinical phenotypes; (**C**) Venn diagram showed the intersection of NAR-associated, TRG- associated, and cancer progression(also PFS)-associated gene sets; (**D**) Top 10 pathways of GO enrichment analysis of the turquoise module; (**E**) Top 10 pathways of KEGG functional enrichment of the turquoise module; (**F**) Top 10 pathways of GO enrichment analysis of the brown module; (**G**) Top 10 pathways of KEGG functional enrichment of the brown module LARC: locally advanced rectal cancer; WGCNA: weighted gene co-expression network analysis; NAR: neoadjuvant rectal; TRG: tumor regression grading; PFS: progression-free survival; GO: Gene Ontology; KEGG: Kyoto Encyclopedia of Genes and Genomes

A weighted gene co-expression network was constructed to identify NCRT-regulated genes (Fig. 2A). A total of 3 modules were identified by merging the high similarity modules. Here, as seen in Fig. 2B, a negative correlation of the brown module with TRG was present (r=-0.24, *P* = 0.06), and the blue module positively correlated with carcinoembryonic antigen (CEA, r = 0.25, *P* = 0.05). The turquoise module (r = 0.28, *P* = 0.02) had the highest positive correlation with the NAR score, while the brown module (r=-0.47, *P* < 0.01) with the highest negative correlation. Next, we also identified 1147 TRG-associated genes and 692 cancer progression genes(PFS-associated genes), respectively (all *P* < 0.05). The intersection among the three sets was estimated to determine the candidate genes with the predictive capabilities of both pathological response and prognosis. Finally, there were five genes overlapped among three sets, including FBXO7, CCT5, ELF1, GSTT4, and SLC44A1, which were identified as the candidate genes (Fig. 2C). In addition, GO and KEGG pathway analyses were performed for the turquoise module (Fig. 2D and E) and the brown module (Fig. 2F and G) genes to gain a more comprehensive understanding of biological effects.

### Internal validation of predictive capabilities of candidate genes

**Fig. 3:**
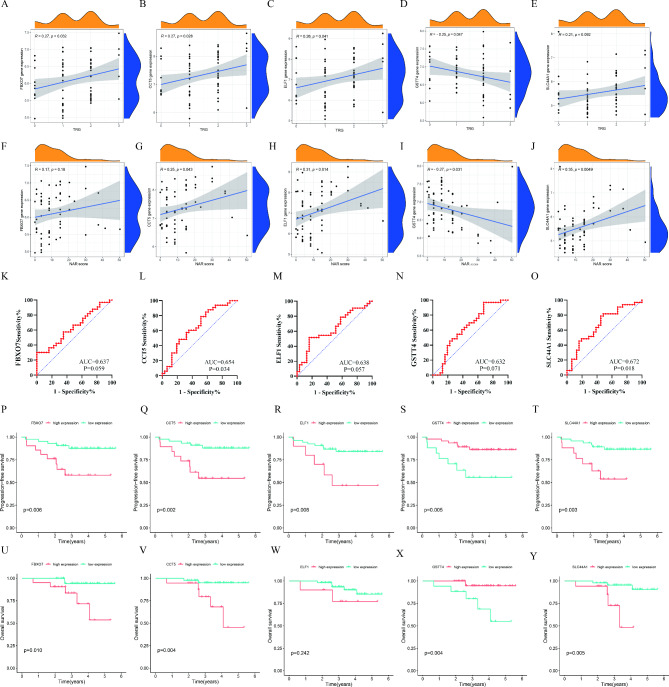
**Internal validation of candidate genes** In 64 LARC patients, the correlations between FBXO7 (**A**), CCT5 (**B**), ELF1 (**C**), GSTT4 (**D**), and SLC44A1 (**E**) expressions and TRG grade; The association between FBXO7 (**F**), CCT5 (**G**), ELF1 (**H**), GSTT4 (**I**), and SLC44A1 (**J**) expressions and NAR score; ROC analysis for the expression of FBXO7 (**K**), CCT5 (L), ELF1 (**M**), GSTT4 (**N**), and SLC44A1 (**O**) to predict NCRT response; KM survival curves for PFS (**P**-**Q**) and OS (**U**-**Y**) of FBXO7, CCT5, ELF1, GSTT4, and SLC44A1. LARC: locally advanced rectal cancer; TRG: tumor regression grading; NAR: neoadjuvant rectal; NCRT: neoadjuvant chemoradiotherapy; KM: Kaplan − Meier; ROC: receiver operating characteristics; AUC: the area under the curve

Next, to test the discriminant power of candidate genes in NCRT sensitivity, the correlations between candidate genes’ expression and TRG grade were calculated. The R-values of the correlation for FBXO7, CCT5, ELF1, GSTT4, and SLC44A1 were 0.27 (*P* = 0.032), 0.27 (*P* = 0.028), 0.26 (*P* = 0.041), -0.25 (*P* = 0.047), and 0.21 (*P* = 0.092), respectively (Fig. 3A and E). We also evaluated the association between candidate genes and NAR score, and the results revealed a significant correlation between these (Fig. 3F J). Next, we employed the gene expression of five candidate genes to predict the pathologic response of NCRT. As shown in Fig. 3K and O, the AUC values of FBXO7, CCT5, ELF1, GSTT4, and SLC44A1 to predict NCRT response were 0.637 (*P* = 0.059), 0.654 (*P* = 0.034), 0.638 (*P* = 0.057), 0.632 (*P* = 0.071), and 0.672 (*P* = 0.018), respectively. Furthermore, the ability to assess the prognosis of five candidate genes was further explored. The cut-off points of gene expression were determined by X-tile software for survival analysis (Supplementary Fig. 1). KM analysis revealed the candidate genes expression at diagnosis could predict the survival of LARC patients undergoing NRCT. Specifically, high expression of FBXO7, CCT5, ELF1, and SLC44A1 were correlated with poor survival, while high expression of GSTT4 was associated with a better prognosis (Fig. 3P and Y). Notably, no significant overall survival differences were observed in high ELF1 expression (*P* = 0.242).

### External validation analysis

The GSE3493 and GSE119409 datasets were collected from the GEO database for external validation. Gene expression was applied to predict the treatment response (NCRT-sensitive). As depicted in Supplementary Fig. 2A-2D, in GSE3943 dataset, the AUC of FBXO7, CCT5, ELF1 and GSTT4 were 0.636 (*P* = 0.18), 0.512 (*P* = 0.91), 0.697(*P* = 0.05), and 0.575 (*P* = 0.46), respectively. As displayed in Supplementary Fig. 2E-2I, in GSE119409 dataset, CCT5 showed the highest power (AUC = 0.689, *P* = 0.03), followed by FBXO7 (AUC = 0.593, *P* = 0.29), SLC44A1 (AUC = 0.586, *P* = 0.33) GSTT4 (AUC = 0.527, *P* = 0.76) and ELF1 (AUC = 0.515, *P* = 0.86). Furthermore, we also evaluated the effects of candidate genes on clinical outcomes. Based on X-tile, candidate genes were separated as the high and low expression groups in GSE133057. We observed that patients of the CCT5 high expression groups had significantly worse prognoses (*P* = 0.012) and that a trend toward poor survival in the high expression of FBXO7, ELF1, GSTT4, SLC44A1 (all *P* > 0.05,Supplementary Fig. 2J-2 N). However, due to SLC44A1 of GSE3493 being missing, we were unable to verify its performance.

### Immunohistochemistry validation of candidate genes

**Fig. 4:**
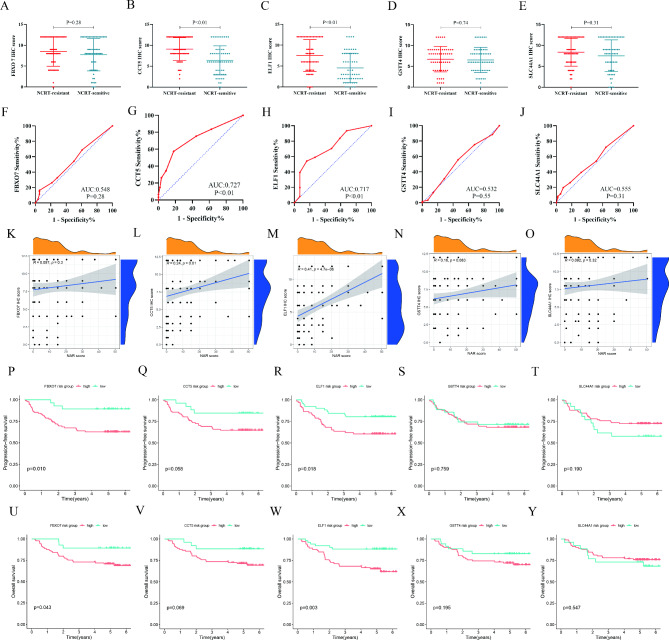
**Immunohistochemistry validation analysis** In the validation cohort of 117 LARC patients, immunohistochemistry score of FBOX7 (**A**), CCT5 (**B**), ELF1 (**C**), GSTT4 (**D**), and SLC44A1 (**E**) between NCRT-sensitive and NCRT-resistant patients, ROC analysis for candidate genes to predict NCRT response (**F**-**J**), the correlation between FBOX7 (K), CCT5 (L), ELF1 (M), GSTT4 (N), and SLC44A1 (O) and NAR score, KM survival curves for PFS (**P**-**Q**) and OS (**U**-**Y**) of FBXO7, CCT5, ELF1, GSTT4, and SLC44A1. NCRT: neoadjuvant chemoradiotherapy; ROC: receiver operating characteristics; AUC: the area under the curve; KM: Kaplan − Meier

To further validate the gene expression of five candidate genes in LARC patients undergoing NCRT, a total of 117 patients were enrolled in the validation cohort. Clinicopathological features of the validation cohort were shown in Supplementary Table 2. We assessed the protein expression of five candidate genes by immunohistochemical staining in tumor biopsy samples from these patients before NCRT. Supplementary Fig. 3 illustrated the different expression levels of five genes. The result demonstrated a higher immunohistochemistry score of CCT5 and ELF1 in NCRT-resistant patients compared with NCRT-sensitive patients (all *P* < 0.05,Fig. 4B C), while no difference was observed in the expression of FBXO7, SLC44A1, and GSTT4 (Fig. 4A, D and E). The ROC curve indicated the AUC of CCT5 and ELF1 to predict the treatment response to NCRT were 0.727 and 0.717 (Fig. 4G H, all *P* < 0.05), and FBXO7, GSTT4, and SLC44A1 cannot differentiate between two groups (Fig. 4F J, all *P* > 0.05). Moreover, CCT5 and ELF1 expression were significantly related to the NAR score (Fig. 4L M), while the trend was not observed in FBXO7, GSTT4, and SLC44A1 (Fig. 4K N-4O). Survival analysis demonstrated that high expression of FBXO7, CCT5, and ELF1 had worse PFS (*P* = 0.010, *P* = 0.058, *P* = 0.018, respectively, Fig. 4P and R) and OS (*P* = 0.043, *P* = 0.069, *P* = 0.003, respectively,Fig. 4U W). At the same time, there was no difference in survival between high and low GSTT4 and SLC44A1 expression, (Fig. 4S, T, X and Y).

### Construction of a risk score model

**Fig. 5:**
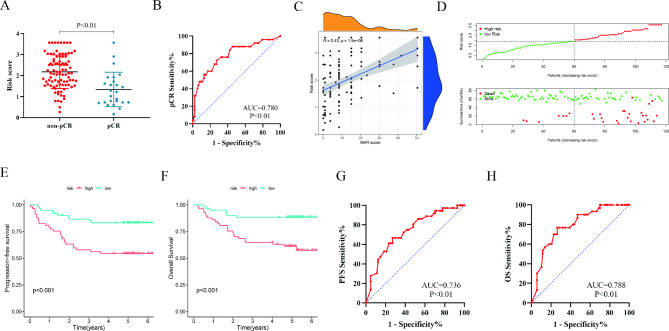
**Development of a risk score model** In the validation cohort of 117 LARC patients, the risk score between non-pCR and pCR patients (**A**) and the capacities of the risk score to predictive pCR (**B**), the relationship between risk score and NAR (**C**) and survival (**D**), KM analysis between high-risk and low-risk groups in PFS (**E**) and OS (**F**), ROC analysis for the risk score to predict PFS (**G**) and OS (**H**). LARC: locally advanced rectal cancer; pCR: pathological complete response; NAR: neoadjuvant rectal; KM: Kaplan − Meier; ROC: receiver operating characteristics; AUC: the area under the curve; PFS: progression-free survival; OS: overall survival

We further performed Cox analysis to determine the prognostic value of genes. On univariate analysis, a high CCT5 and ELF1 expression were associated with shorter OS (all *P* < 0.05). Multivariate analysis revealed that CCT5 (hazard ratio [HR], 1.141, 95% confidence interval [CI]: 1.007–1.294, *P* = 0.039) and ELF1 (HR,1.179, 95%CI, 1.067–1.304, *P* = 0.001) were independently associated with prognosis (Table 1). Hence, based on coefficients obtained from Cox analysis, the risk score was computed as: 0.132×CCT5 expression + 0.165×ELF1 expression. We divided the validation cohort into two groups based on the median risk score, and the clinicopathologic characteristics of the two groups were presented in Supplementary Table 3. The risk score of the pathological complete response (pCR) group was significantly lower than the non-pCR group (Fig. 5A), with excellent predictive capacity (AUC = 0.780, *P* < 0.01, Fig. 5B). A significant correlation was observed between the risk score and NAR score (R = 0.43, *P* < 0.01, Fig. 5C). Survival analysis showed the risk score has an excellent ability to discriminate clinical outcomes (Fig. 5D H).

**Table 1:** Cox analysis of OS for candidate gene in the validation cohort of 117 LARC patietns

Characteristics	Univariate				Multivariate		
	HR	95% CI	*P* value	β	HR	95% CI	*P* value
FBXO7 expression	1.089	0.979–1.211	0.115				
CCT5 expression	1.200	1.062–1.355	0.003	0.132	1.141	1.007–1.294	0.039
ELF1 expression	1.215	1.101–1.341	< 0.001	0.165	1.179	1.067–1.304	0.001
GSTT4 expression	1.090	0.967–1.229	0.157				
SLC44A1 expression	1.017	0.918–1.127	0.749				

OS: overall survival; LARC: locally advanced rectal cancer; NCRT: neoadjuvant chemoradiotherapy

### Prognosis analyses and development nomogram

**Fig. 6:**
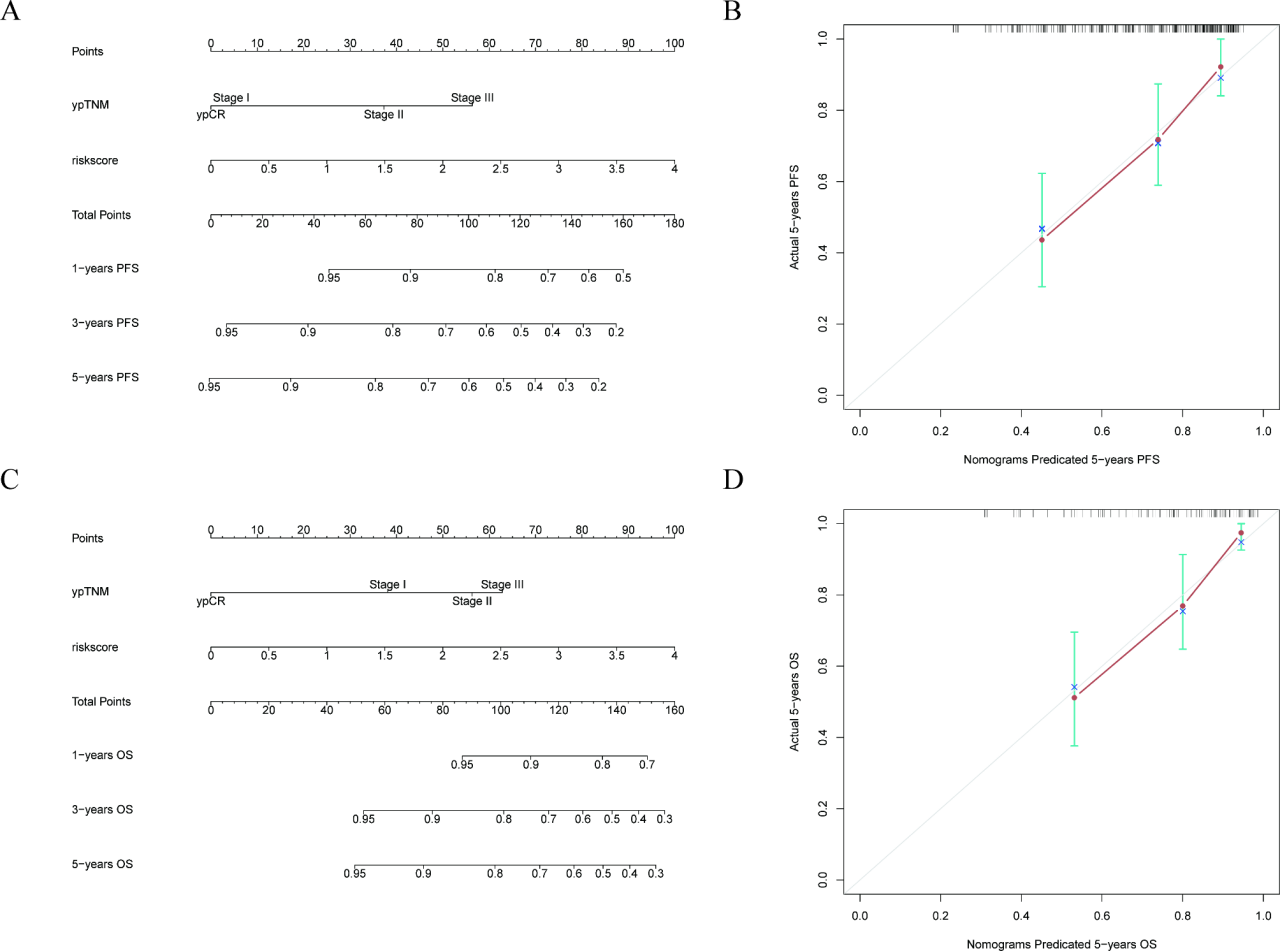
**Construction nomograms for PFS and OS.** In the validation cohort of 117 LARC patients, nomogram to predict PFS (**A**) and OS (**B**) for LARC patients undergoing NCRT, the calibration curve was used for model validation for PFS (**C**) and OS (**D**). PFS: progression-free survival; OS: overall survival; LARC: locally advanced rectal cancer; NCRT: neoadjuvant chemoradiotherapy

We after that evaluated the prognostic value of the risk score. Univariate analysis revealed that the ypTMN stage (*P* < 0.001), TRG (*P* < 0.001), pathological type (*P* = 0.036), risk score (*P* < 0.001), and NAR score (*P* = 0.006) were significantly associated with PFS. Multivariate analysis manifested that the ypTMN stage ( *P* = 0.040) and risk score ( *P* = 0.025) were independent risk factors of PFS (Table 2). Furthermore, as shown in Table 3, the ypTMN stage ( *P* = 0.031) and risk score ( *P* = 0.008) were significantly correlated with OS by multivariate analysis. Results of the analysis based on Cox analysis, nomograms were constructed to predict 1-year, 3-year-, and 5-year PFS (Fig. 6A) and OS (Fig. 6C), and good calibration was also confirmed (Fig. 6B and D).

**Table 2:** Univariate and multivariable Cox analyses of PFS in the validation cohort of 117 LARC patients

Characteristics	Univariate			Multivariate		
	HR	95% CI	*P* value	HR	95% CI	*P* value
Age, years	1.007	0.977–1.038	0.645			
Sex: female versus male	1.214	0.621–2.373	0.570			
ASA	1.102	0.603–2.015	0.752			
Distance from the anal verge	1.012	0.898–1.141	0.845			
Pre-NCRT CEA: >5 ng/ml versus ≤ 5 ng/ml	1.467	0.762–2.823	0.252			
Pre-NCRT CA19-9: >37 U/ml versus ≤ 37 U/ml	1.788	0.744–4.298	0.194			
Interval time between NCRT and surgery	1.057	0.893–1.250	0.520			
ypTMN stage	2.044	1.425–2.931	< 0.001	1.943	1.031–3.661	0.040
TRG	2.133	1.408–3.232	< 0.001	1.367	0.774–2.413	0.281
Pathological type: MAC or SRCC versus adenocarcinoma	2.428	1.062–5.549	0.036	1.261	0.495–3.207	0.627
Risk score	2.305	1.548–3.433	< 0.001	1.705	1.070–2.719	0.025
NAR score	1.030	1.009–1.052	0.006	0.971	0.931–1.013	0.172

PFS: progression-free survival; LARC: locally advanced rectal cancer; NCRT: neoadjuvant chemoradiotherapy; ASA: American society of anesthesiologists; CEA: carcinoembryonic antigen; CA19-9: carbohydrate antigen 19 − 9; TNM: tumor-node-metastasis; TRG: tumor regression grading; MAC: mucinous adenocarcinoma; SRCC: signet ring cell carcinoma; NAR score: neoadjuvant rectal-score

**Table 3:** Univariate and multivariable Cox analyses of OS in the validation cohort of 117 LARC patients

Characteristics	Univariate			Multivariate		
	HR	95% CI	*P* value	HR	95% CI	*P* value
Age, years	1.008	0.975–1.041	0.647			
Sex: female versus male	1.308	0.630–2.719	0.471			
ASA	0.983	0.493–1.959	0.960			
Distance from the anal verge	1.007	0.882–1.149	0.919			
Pre-NCRT CEA: >5 ng/ml versus ≤ 5 ng/ml	1.220	0.592–2.513	0.590			
Pre-NCRT CA19-9: >37 U/ml versus ≤ 37 U/ml	1.390	0.485–3.985	0.540			
Interval time between NCRT and surgery	1.013	0.839–1.223	0.891			
ypTMN stage	2.181	1.453–3.272	< 0.001	2.174	1.073–3.814	0.031
TRG	2.193	1.392–3.455	0.001	1.247	0.661–2.351	0.495
Pathological type: MAC or SRCC versus adenocarcinoma	2.955	1.266–6.896	0.012	1.487	0.579–3.814	0.409
Risk score	2.719	1.737–4.257	< 0.001	2.036	1.201–3.452	0.008
NAR score	1.031	1.008–1.056	0.009	0.962	0.918–1.009	0.108

OS: overall survival; LARC: locally advanced rectal cancer; NCRT: neoadjuvant chemoradiotherapy; ASA: American society of anesthesiologists; CEA: carcinoembryonic antigen; CA19-9: carbohydrate antigen 19 − 9; TNM: tumor-node-metastasis; TRG: tumor regression grading; MAC: mucinous adenocarcinoma; SRCC: signet ring cell carcinoma; NAR score: neoadjuvant rectal-score

### External validation for risk score

The risk score of the NCRT-resistant group was considerably higher in the GSE3493 cohort than the NCRT-sensitive group ( Supplementary Fig. 4A) with an adequate capacity to discriminate NCRT-sensitive patients (AUC = 0.701, *P* = 0.046, Supplementary Fig. 4C). However, we found no statistical difference between the gene expression of the GSE119409 cohort ( Supplementary Fig. 4B, 4D) and the prognosis of the GSE133057 cohort (all *P* > 0.05, Supplementary Fig. 4E).

### GSEA analysis

**Fig. 7:**
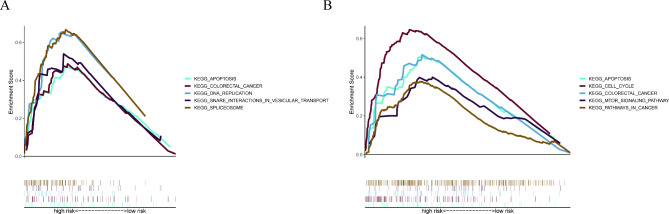
**GSEA analysis** In 64 LARC patients, potential biological pathways are enriched in the high CCT5 expression group (**A**) and the high ELF1 expression group (**B**) LARC: locally advanced rectal cancer

Following that, we look into the potential mechanisms by which CCT5 and ELF1 might impact NCRT sensitivity. The median expression level defined the groups with high and low CCT5 and ELF1 expression. Colorectal cancer, apoptosis, and DNA replication were enriched in the high CCT5 expression group, according to GSEA analysis (Fig. 7A). Furthermore, the high ELF1 expression group was significantly enriched with apoptosis, cell cycle, mTOR signaling pathway, and cancer pathway (Fig. 7B). These pathways contribute to a better understanding of the mechanism of NCRT resistance.

#### Drug sensitivity analysis

**Fig. 8:**
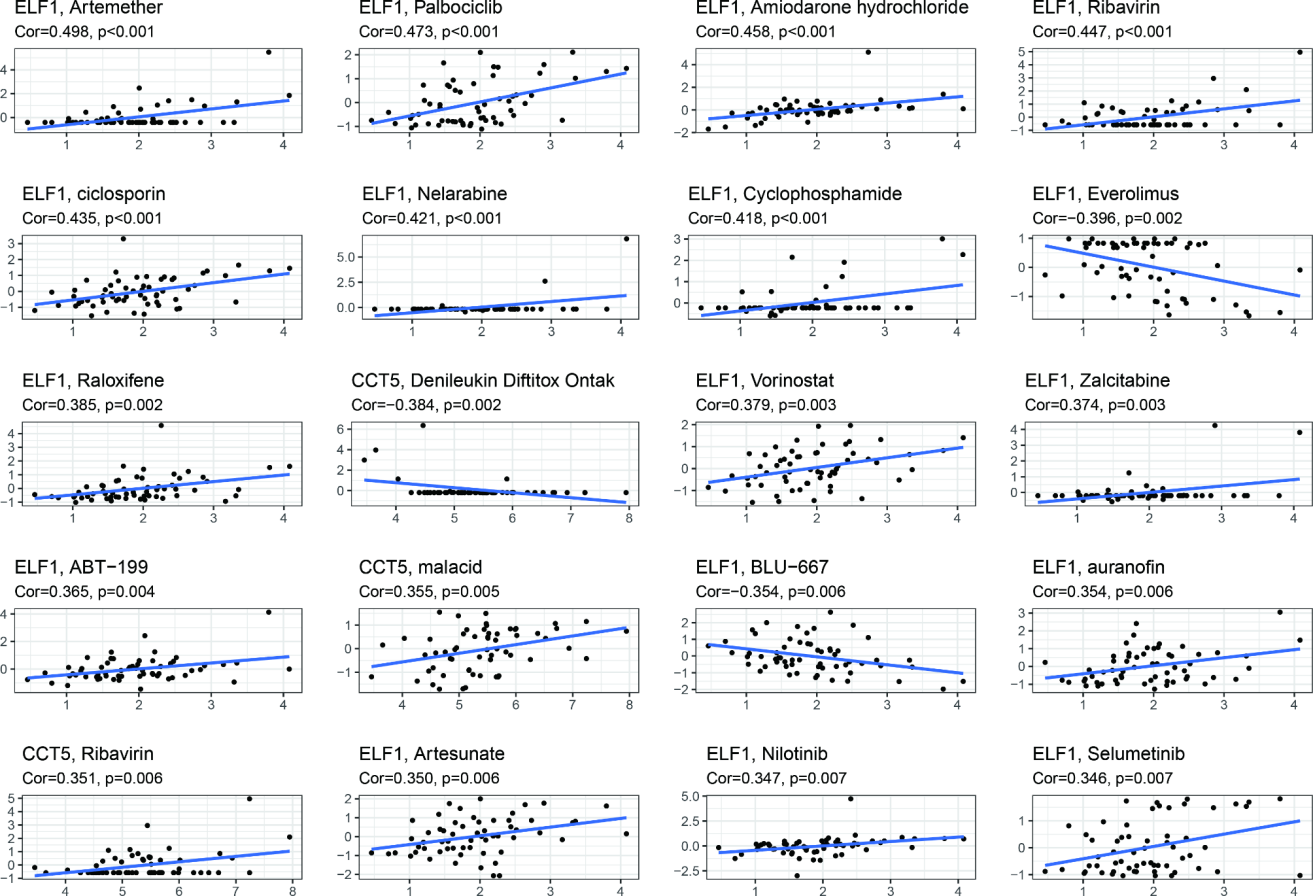
**Drug sensitivity analysis** The relationship between CCT5 and ELF1 and drug sensitivity using the CellMiner database. The x-axis represents the gene expression, and the y-axis is drug sensitivity

We then examined the association of CCT5 and ELF1 with drug sensitivity using the CellMiner database. Figure 8 demonstrated the results of the drug sensitivity analysis. For instance, the ELF1 expression was positively associated with the drug sensitivity of vorinostat, artesunate, nilotinib, and selumetinib (All *P* < 0.05).

### Analysis of immune infiltration

**Fig. 9:**
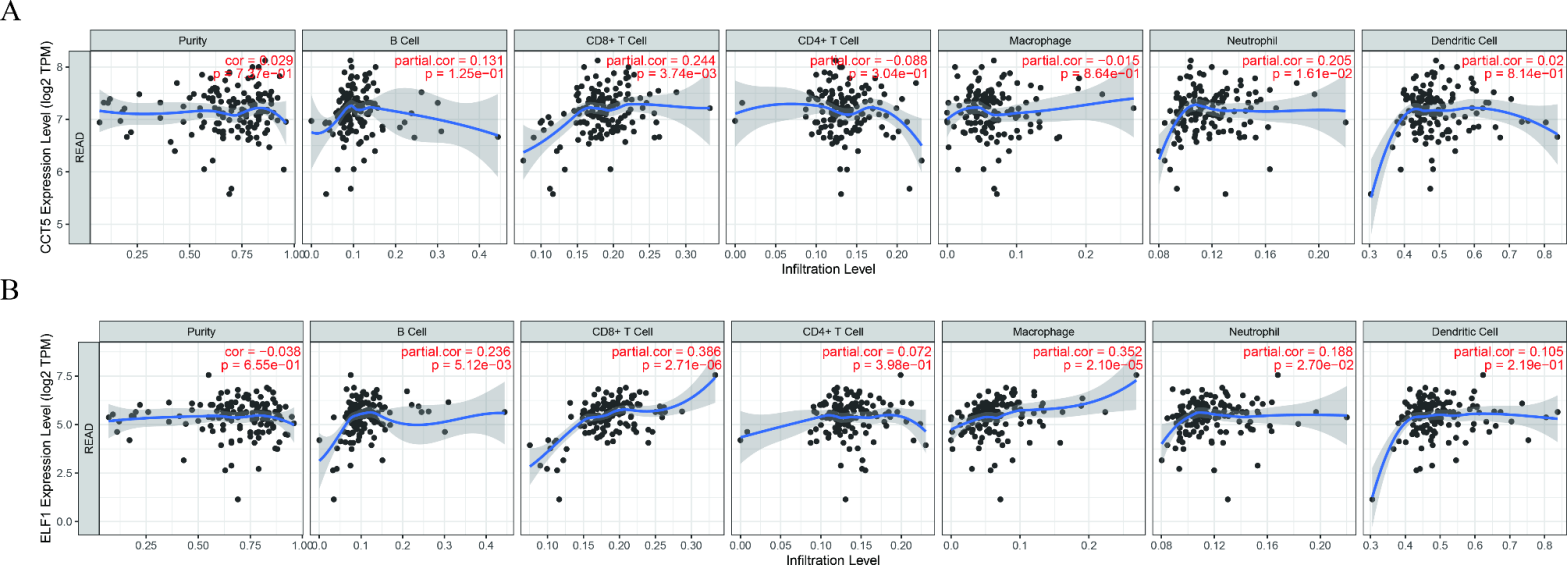
**Analysis of immune infiltration** The correlations between CCT5 (**A**), ELF1 (**B**) expression, and immune cell infiltration using Tumor Immune Estimation Resource database

Next, we also investigated immune cell infiltration. As shown in Fig. 9A, positive correlations were observed between CCT5 expression and the infiltration of CD8 + T cell (*P* < 0.01) and neutrophil (*P* < 0.05). B cell, CD8 + T cell, macrophage, and neutrophil infiltration were significantly associated with ELF1 expression (All *P* < 0.05, Fig. 9B).

## Discussion

Previous studies have demonstrated the superiority of NCRT in LARC treatment. At the moment, the mechanism of NCRT resistance remains unclear. Given treatment resistance in certain patients and potential complications and toxicity, further research is warranted. The expression profile of the pre-treatment tumor sample from our cohort was used for the analyses in this study. WGCNA was utilized to determine NAR score-related modules and genes. To determine the potential genes with a high ability to predict sensitivity to NCRT and prognosis, TRG-related and cancer progression (also PFS)-related genes were also identified. The intersection of three gene sets was regarded as candidate genes for additional validation at the protein level. The protein expression of CCT5 and ELF1 were significantly different in the NCRT-sensitive and NCRT-resistant groups according to the results of immunohistochemistry staining of candidate genes. Furthermore, high-level expression of CCT5 and ELF1 were linked to a poor prognosis.

The NAR score has recently been considered a potential surrogate endpoint for the prognosis of LARC patients undergoing NCRT [11]. This score formula was established on the basis of Valentini et al. nomogram for predicting the overall survival in LARC patients, and it considered both pretreatment tumor burden and posttreatment tumor regression [17]. Previous researches have demonstrated that the NAR score can accurately predict clinical outcomes and assist in assessing the necessity of adjunctive therapy [18–20]. Thus, we found NAR score-related genes using WCGNA, a more biological approach to correlating modules with phenotypic features. However, there is some debate as to the prognostic value of the NAR score. A study from the Netherlands demonstrated that a combination model based on clinical data and pathological data exceeded the NAR score in assessing survival outcomes [21]. As a result, cancer progression (also PFS)-associated genes were also discovered. Therefore, discerning therapy response before the surgical procedure aided clinical decision-making. After adequate evaluation, Watch & Wait and local excision might become a suitable therapy for NCRT-sensitive patients, maximizing the patient and enhancingn appropriat the quality of life [22]. Hence, we identify TRG-associated genes, and the intersection of three gene sets contributes to determining the genes with the value of predicting prognosis and treatment response.

FBXO7, CCT5, ELF1, GSTT4, and SLC44A1 were discovered as biomarkers for treatment response and prognosis in our cohort. The value of CCT5 and ELF1 in predicting response to therapy was validated in the GSE3493 and GSE119409 cohorts. The survival analysis of the GSE133057 cohort showed that increased expression of FBXO7, CCT5, ELF1, and SLC44A1 implies poor survival, whereas high expression of GSTT4 predicts the opposite outcomes. These survival trends match those obtained in our cohort. CCT5 and ELF1 were eventually determined after the IHC validation of protein levels.

CCT5 is a protein folding subunit of the chaperonin containing TCP1 complex (also known as TCP1-ring complex). Recently, accumulating evidence reveals that CCT5 was implicated in tumor progression, and high expression of CCT5 has been discovered in a range of malignancies and associated with worse survival [23–25]. Notably, CCT5 is involved in chemotherapy resistance. In breast cancer, the knockdown of CCT5 leads to increased apoptosis after docetaxel treatment [26]. CCT5 expression was elevated in multidrug-resistant gastric carcinoma cells [27]. Nevertheless, the functions of CCT5 in NCRT of LARC patients remain elusive. Our studies demonstrate that high CCT5 expression correlated with NAR score, NCRT resistance, and poor prognosis in LARC patients.

ELF1 is a transcription factor that belongs to the ETS family and plays contrasting roles in different tumors. Some reports pointed out the part of ELF1 has tumor-promoting effects in glioma [28, 29], oral squamous cell carcinoma [30], choroidal melanoma [31], endometrial carcinoma [32], acute myeloid leukemia [33], nasopharyngeal carcinoma [34], and osteosarcoma [35], while others have reported a tumor-suppressive function in Hodgkin lymphoma [36] and prostate cancer [37]. Starr et al. found that ELF1 regulates the expression of TM9SF2, an oncogene in colorectal cancer [38]. Herein, our findings indicated that ELF1 expression was more strongly raised in NCRT-resistant patients, and ELF1 was an independent predictor of survival. Going further, GSEA analysis revealed apoptosis, cell cycle, and mTOR signaling pathway are significantly enriched in high ELF1 expression, and those pathways were implicated in treatment resistance [39–41].

Compared to a single prognostic factor, the advantages of the risk model and nomogram have previously been reported [42, 43]. In our study, we build a risk score model based on CCT5 and ELF1 IHC expression that effectively discriminated between non-pCR and pCR patients and survival. Nomograms were created by taking together the result of Cox regression analysis. These models are helpful in predicting the prognosis of LARC patients undergoing NCRT.

Furthermore, a growing body of studies has revealed the impact of tumor microenvironment on chemoradiotherapy sensitivity. One study reported that increased CD163 + tumor-associated macrophages (TAM) were observed in non-pCR group in LARC patients [44]. Meanwhile, TAM infiltration was confirmed with chemoradiotherapy resistance in oral squamous cell carcinoma and cervical cancer [45, 46]. In addition, several previous studies have revealed that increased neutrophils were associated with poor chemoradiotherapy response and worse clinical outcomes [47–50]. Here, our study also explored the potential role of CCT5 and ELF1 in the tumor microenvironment and found the infiltration level of CD8 + T cell and neutrophil were positively associated with CCT5 and ELF1 expression, and the same trend was observed in the correlation between B cell and macrophage and ELF1 expression. However, there are limited studies about CCT5 or ELF1 leading to immune infiltration, and the specific mechanism remains to be elucidated. Nonetheless, these findings provided valuable information and potential directions for future research.

There are limitations to this study. More external datasets are required to confirm and validate our findings. Meanwhile, bioinformatics has deconstructed the probable processes of CCT5 and ELF1, but further investigation will be needed to clarify their biological role and impact on the cancer microenvironment in future studies.

## Conclusion

In summary, CCT5 and ELF1 were determined as biomarkers for NCRT treatment response and prognosis by internal and external validation. The risk score model and nomogram were constructed to predict survival. These findings contributed to personalized clinical decision-making for LARC patients undergoing NCRT.

### Electronic supplementary material

Below is the link to the electronic supplementary material.

Supplementary Material 1

Supplementary Material 2

Supplementary Material 3

Supplementary Material 4

Supplementary Material 5

Supplementary Material 6

Supplementary Material 7

Supplementary Material 8

## Data Availability

External validation datasets (GSE3493, GSE119409, and GSE133057) were obtained from Gene Expression Omnibus (http://www.ncbi.nlm.nih.gov/geo/). The datasets generated during and/or analyzed during the current study are available from the corresponding author on reasonable request.

## References

[1] Sung H, Ferlay J, Siegel RL, Laversanne M, Soerjomataram I, Jemal A, Bray F (2021). Global Cancer Statistics 2020: GLOBOCAN estimates of incidence and Mortality Worldwide for 36 cancers in 185 countries. CA Cancer J Clin.

[2] Siegel RL, Miller KD, Goding Sauer A, Fedewa SA, Butterly LF, Anderson JC, Cercek A, Smith RA, Jemal A (2020). Colorectal cancer statistics, 2020. CA Cancer J Clin.

[3] Li Y, Wang J, Ma X, Tan L, Yan Y, Xue C, Hui B, Liu R, Ma H, Ren J (2016). A review of Neoadjuvant Chemoradiotherapy for locally advanced rectal Cancer. Int J Biol Sci.

[4] Ominelli J, Valadão M, Araujo ROC, Cristina de Melo A, Araujo LH (2021). The Evolving Field of Neoadjuvant Therapy in locally-advanced rectal Cancer: evidence and prospects. Clin Colorectal Cancer.

[5] Gollins S, Sebag-Montefiore D (2016). Neoadjuvant treatment strategies for locally advanced rectal Cancer. Clin Oncol (R Coll Radiol (G B)).

[6] Ha YJ, Tak KH, Kim CW, Roh SA, Choi EK, Cho DH, Kim JH, Kim SK, Kim SY, Kim YS (2017). PSMB8 as a candidate marker of responsiveness to Preoperative Radiation Therapy in rectal Cancer patients. Int J Radiat Oncol Biol Phys.

[7] Seo I, Lee HW, Byun SJ, Park JY, Min H, Lee SH, Lee JS, Kim S, Bae SU. Neoadjuvant chemoradiation alters biomarkers of anticancer immunotherapy responses in locally advanced rectal cancer. J Immunother Cancer 2021, 9(3). 10.1136/jitc-2020-001610 PMC7949478 33692216

[8] Momma T, Okayama H, Kanke Y, Fukai S, Onozawa H, Fujita S, Sakamoto W, Saito M, Ohki S, Kono K. Validation of Gene Expression-Based Predictive Biomarkers for Response to Neoadjuvant Chemoradiotherapy in Locally Advanced Rectal Cancer. *Cancers (Basel)* 2021, 13(18). 10.3390/cancers13184642 PMC8467397 34572869

[9] Agostini M, Zangrando A, Pastrello C, D’Angelo E, Romano G, Giovannoni R, Giordan M, Maretto I, Bedin C, Zanon C (2015). A functional biological network centered on XRCC3: a new possible marker of chemoradiotherapy resistance in rectal cancer patients. Cancer Biol Ther.

[10] Millino C, Maretto I, Pacchioni B, Digito M, De Paoli A, Canzonieri V, D’Angelo E, Agostini M, Rizzolio F, Giordano A (2017). Gene and MicroRNA expression are Predictive of Tumor response in rectal adenocarcinoma patients treated with preoperative chemoradiotherapy. J Cell Physiol.

[11] Glynne-Jones R, Glynne-Jones S (2021). The concept and use of the neoadjuvant rectal score as a composite endpoint in rectal cancer. Lancet Oncol.

[12] Fokas E, Fietkau R, Hartmann A, Hohenberger W, Grützmann R, Ghadimi M, Liersch T, Ströbel P, Grabenbauer GG, Graeven U (2018). Neoadjuvant rectal score as individual-level surrogate for disease-free survival in rectal cancer in the CAO/ARO/AIO-04 randomized phase III trial. Ann Oncol.

[13] Li A, Huang T, Zheng R, Chi P, Li Z, Wang X, Xu B (2022). Preoperative chemoradiotherapy with capecitabine and triweekly oxaliplatin versus capecitabine monotherapy for locally advanced rectal cancer: a propensity-score matched study. BMC Cancer.

[14] Langfelder P, Horvath S (2008). WGCNA: an R package for weighted correlation network analysis. BMC Bioinformatics.

[15] Feng C, Zhang Y, Huang J, Zheng Q, Yang Y, Xu B (2021). The Prognostic significance of APOBEC3B and PD-L1/PD-1 in nasopharyngeal carcinoma. Appl Immunohistochem Mol Morphology: AIMM.

[16] Shankavaram UT, Varma S, Kane D, Sunshine M, Chary KK, Reinhold WC, Pommier Y, Weinstein JN (2009). CellMiner: a relational database and query tool for the NCI-60 cancer cell lines. BMC Genomics.

[17] Valentini V, van Stiphout RG, Lammering G, Gambacorta MA, Barba MC, Bebenek M, Bonnetain F, Bosset JF, Bujko K, Cionini L (2011). Nomograms for predicting local recurrence, distant metastases, and overall survival for patients with locally advanced rectal cancer on the basis of european randomized clinical trials. J Clin Oncol.

[18] Lim YJ, Song C, Jeon SH, Kim K, Chie EK (2021). Risk stratification using neoadjuvant rectal score in the era of Neoadjuvant Chemoradiotherapy: Validation with Long-term Outcome Data. Dis Colon Rectum.

[19] Huang WS, Kuan FC, Lin MH, Chen MF, Chen WC (2020). Prognostic significance of Neoadjuvant rectal scores in Preoperative Short-Course Radiotherapy and Long-Course Concurrent Chemoradiotherapy for patients with locally advanced rectal Cancer. Ann Surg Oncol.

[20] Roselló S, Frasson M, García-Granero E, Roda D, Jordá E, Navarro S, Campos S, Esclápez P, García-Botello S, Flor B (2018). Integrating downstaging in the Risk Assessment of patients with locally advanced rectal Cancer treated with Neoadjuvant Chemoradiotherapy: validation of Valentini’s Nomograms and the neoadjuvant rectal score. Clin Colorectal Cancer.

[21] van der Valk MJM, Vuijk FA, Putter H, van de Velde CJH, Beets GL, Hilling DE (2019). Disqualification of neoadjuvant rectal score based on data of 6596 patients from the Netherlands Cancer Registry. Clin Colorectal Cancer.

[22] Smith JJ, Strombom P, Chow OS, Roxburgh CS, Lynn P, Eaton A, Widmar M, Ganesh K, Yaeger R, Cercek A (2019). Assessment of a Watch-and-wait strategy for rectal Cancer in patients with a complete response after Neoadjuvant Therapy. JAMA Oncol.

[23] Meng Y, Yang L, Wei X, Luo H, Hu Y, Tao X, He J, Zheng X, Xu Q, Luo K (2021). CCT5 interacts with cyclin D1 promoting lung adenocarcinoma cell migration and invasion. Biochem Biophys Res Commun.

[24] He J, McLaughlin RP, van der Beek L, Canisius S, Wessels L, Smid M, Martens JWM, Foekens JA, Zhang Y, van de Water B (2020). Integrative analysis of genomic amplification-dependent expression and loss-of-function screen identifies ASAP1 as a driver gene in triple-negative breast cancer progression. Oncogene.

[25] Li Y, Liu C, Zhang X, Huang X, Liang S, Xing F, Tian H (2022). CCT5 induces epithelial-mesenchymal transition to promote gastric cancer lymph node metastasis by activating the Wnt/β-catenin signalling pathway. Br J Cancer.

[26] Ooe A, Kato K, Noguchi S (2007). Possible involvement of CCT5, RGS3, and YKT6 genes up-regulated in p53-mutated tumors in resistance to docetaxel in human breast cancers. Breast Cancer Res Treat.

[27] Ludwig A, Dietel M, Lage H (2002). Identification of differentially expressed genes in classical and atypical multidrug-resistant gastric carcinoma cells. Anticancer Res.

[28] Cheng M, Zeng Y, Zhang T, Xu M, Li Z, Wu Y (2021). Transcription factor ELF1 activates MEIS1 transcription and then regulates the GFI1/FBW7 Axis to promote the development of Glioma. Mol Therapy Nucleic Acids.

[29] Hu M, Li H, Xie H, Fan M, Wang J, Zhang N, Ma J, Che S (2021). ELF1 transcription factor enhances the progression of Glioma via ATF5 promoter. ACS Chem Neurosci.

[30] Qiao C, Qiao T, Yang S, Liu L, Zheng M (2022). SNHG17/miR-384/ELF1 axis promotes cell growth by transcriptional regulation of CTNNB1 to activate Wnt/β-catenin pathway in oral squamous cell carcinoma. Cancer Gene Ther.

[31] Wang L, Tang D, Wu T, Sun F (2020). ELF1-mediated LUCAT1 promotes choroidal melanoma by modulating RBX1 expression. Cancer Med.

[32] Takai N, Miyazaki T, Nishida M, Shang S, Nasu K, Miyakawa I (2003). Clinical relevance of Elf-1 overexpression in endometrial carcinoma. Gynecol Oncol.

[33] Pang Y, Zhao Y, Wang Y, Wang X, Wang R, Liu N, Li P, Ji M, Ye J, Sun T (2020). TNFAIP8 promotes AML chemoresistance by activating ERK signaling pathway through interaction with Rac1. J Exp Clin Cancer Res.

[34] Chen CH, Su LJ, Tsai HT, Hwang CF (2019). ELF-1 expression in nasopharyngeal carcinoma facilitates proliferation and metastasis of cancer cells via modulation of CCL2/CCR2 signaling. Cancer Manage Res.

[35] Wang L (2021). ELF1-activated FOXD3-AS1 promotes the migration, invasion and EMT of osteosarcoma cells via sponging mir-296-5p to upregulate ZCCHC3. J bone Oncol.

[36] Paczkowska J, Soloch N, Bodnar M, Kiwerska K, Janiszewska J, Vogt J, Domanowska E, Martin-Subero JI, Ammerpohl O, Klapper W (2019). Expression of ELF1, a lymphoid ETS domain-containing transcription factor, is recurrently lost in classical Hodgkin lymphoma. Br J Haematol.

[37] Budka JA, Ferris MW, Capone MJ, Hollenhorst PC (2018). Common ELF1 deletion in prostate cancer bolsters oncogenic ETS function, inhibits senescence and promotes docetaxel resistance. Genes & cancer.

[38] Clark CR, Maile M, Blaney P, Hellweg SR, Strauss A, Durose W, Priya S, Habicht J, Burns MB, Blekhman R (2018). Transposon mutagenesis screen in mice identifies TM9SF2 as a novel colorectal cancer oncogene. Sci Rep.

[39] Adamsen BL, Kravik L, De Angelis PM (2009). Cellular response to chemoradiotherapy, radiotherapy and chemotherapy in two colorectal cancer cell lines. Radiat Res.

[40] Suzuki T, Sadahiro S, Tanaka A, Okada K, Saito G, Kamijo A, Akiba T, Kawada S (2015). Predictive markers of chemoradiotherapy for rectal cancer: comparison of biopsy specimens taken before and about 1 week after the start of chemoradiotherapy. Int J Clin Oncol.

[41] Wanigasooriya K, Tyler R, Barros-Silva JD, Sinha Y, Ismail T, Beggs AD. Radiosensitising Cancer using Phosphatidylinositol-3-Kinase (PI3K), protein kinase B (AKT) or mammalian target of Rapamycin (mTOR) inhibitors. Cancers (Basel) 2020, 12(5). 10.3390/cancers12051278 PMC7281073 32443649

[42] Zheng W, Lu Y, Feng X, Yang C, Qiu L, Deng H, Xue Q, Sun K (2021). Improving the overall survival prognosis prediction accuracy: a 9-gene signature in CRC patients. Cancer Med.

[43] Hong T, Cai D, Jin L, Zhang Y, Lu T, Hua D, Wu X (2020). Development and validation of a nomogram to predict survival after curative resection of nonmetastatic colorectal cancer. Cancer Med.

[44] Yang Y, Tian W, Su L, Li P, Gong X, Shi L, Zhang Q, Zhao B, Zhao H (2021). Tumor-infiltrating cytotoxic T cells and Tumor-Associated Macrophages Correlate with the Outcomes of Neoadjuvant Chemoradiotherapy for locally advanced rectal Cancer. Front Oncol.

[45] Matsuoka Y, Yoshida R, Nakayama H, Nagata M, Hirosue A, Tanaka T, Kawahara K, Nakagawa Y, Sakata J, Arita H (2015). The tumour stromal features are associated with resistance to 5-FU-based chemoradiotherapy and a poor prognosis in patients with oral squamous cell carcinoma. APMIS: Acta Pathologica Microbiologica et immunologica Scandinavica.

[46] Lippens L, Van Bockstal M, De Jaeghere EA, Tummers P, Makar A, De Geyter S, Van de Vijver K, Hendrix A, Vandecasteele K, Denys H (2020). Immunologic impact of chemoradiation in cervical cancer and how immune cell infiltration could lead toward personalized treatment. Int J Cancer.

[47] Hu P, Liu Q, Deng G, Zhang J, Liang N, Xie J, Zhang J (2019). Radiosensitivity nomogram based on circulating neutrophils in thoracic cancer. Future Oncol.

[48] Wisdom AJ, Hong CS, Lin AJ, Xiang Y, Cooper DE, Zhang J, Xu ES, Kuo HC, Mowery YM, Carpenter DJ (2019). Neutrophils promote tumor resistance to radiation therapy. Proc Natl Acad Sci U S A.

[49] Schernberg A, Nivet A, Dhermain F, Ammari S, Escande A, Pallud J, Louvel G, Deutsch E (2018). Neutrophilia as a biomarker for overall survival in newly diagnosed high-grade glioma patients undergoing chemoradiation. Clin Translational Radiation Oncol.

[50] Schernberg A, Vernerey D, Goldstein D, Van Laethem JL, Glimelius B, van Houtte P, Bonnetain F, Louvet C, Hammel P, Huguet F (2021). Predictive value of neutrophils count for local Tumor Control after Chemoradiotherapy in patients with locally advanced pancreatic carcinoma. Int J Radiat Oncol Biol Phys.

