# Supplementary figures and images for: Novel biomarkers to predict treatment response and prognosis in locally advanced rectal cancer undergoing neoadjuvant chemoradiotherapy
Source: BMC Cancer. 2023 Nov 12;23:1099. doi: 10.1186/s12885-023-11354-8 (PMC10642053; doi:10.1186/s12885-023-11354-8)

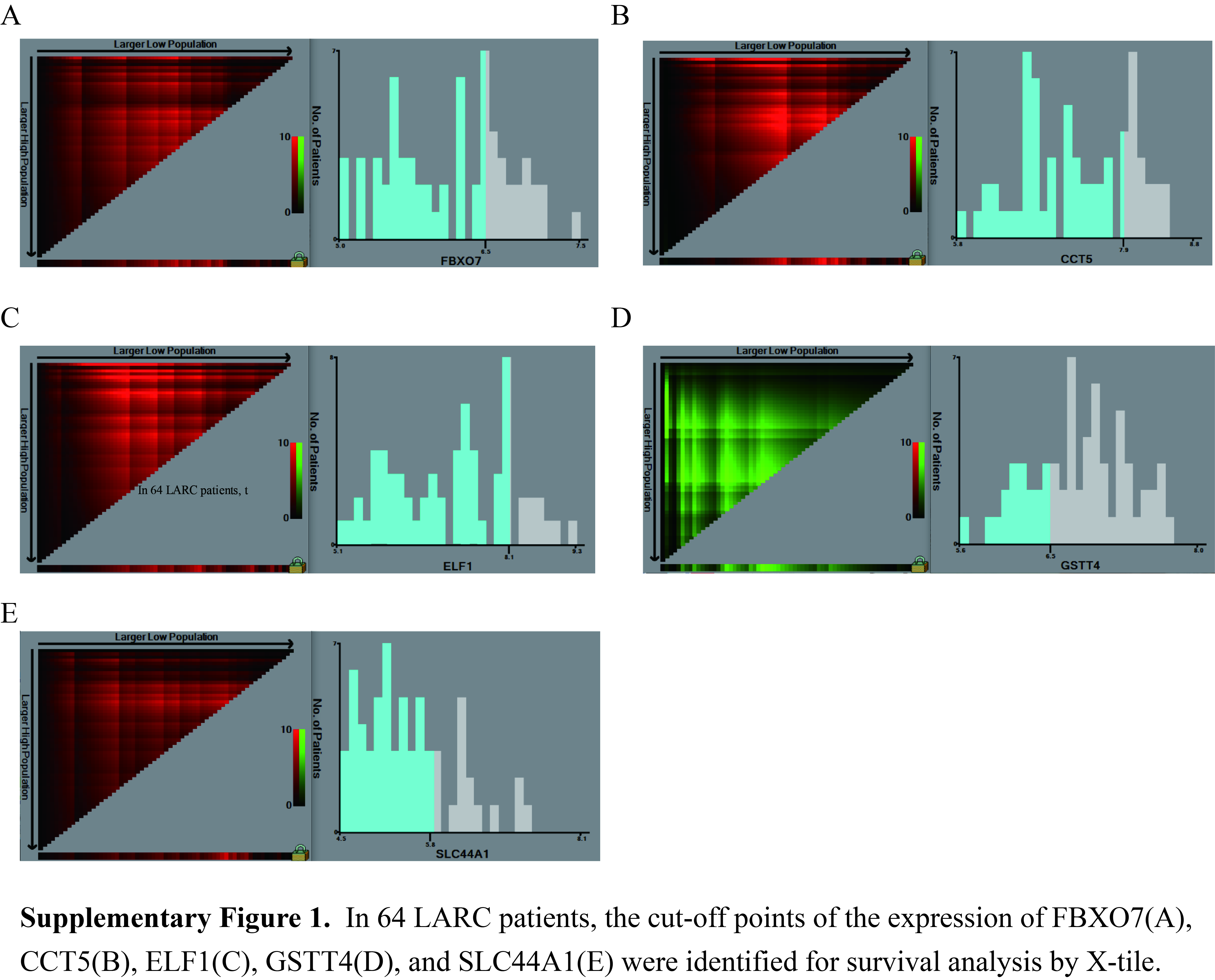

Supplement: Supplementary file 1 — Supplementary Material 1 [file 12885_2023_11354_MOESM1_ESM.tif]

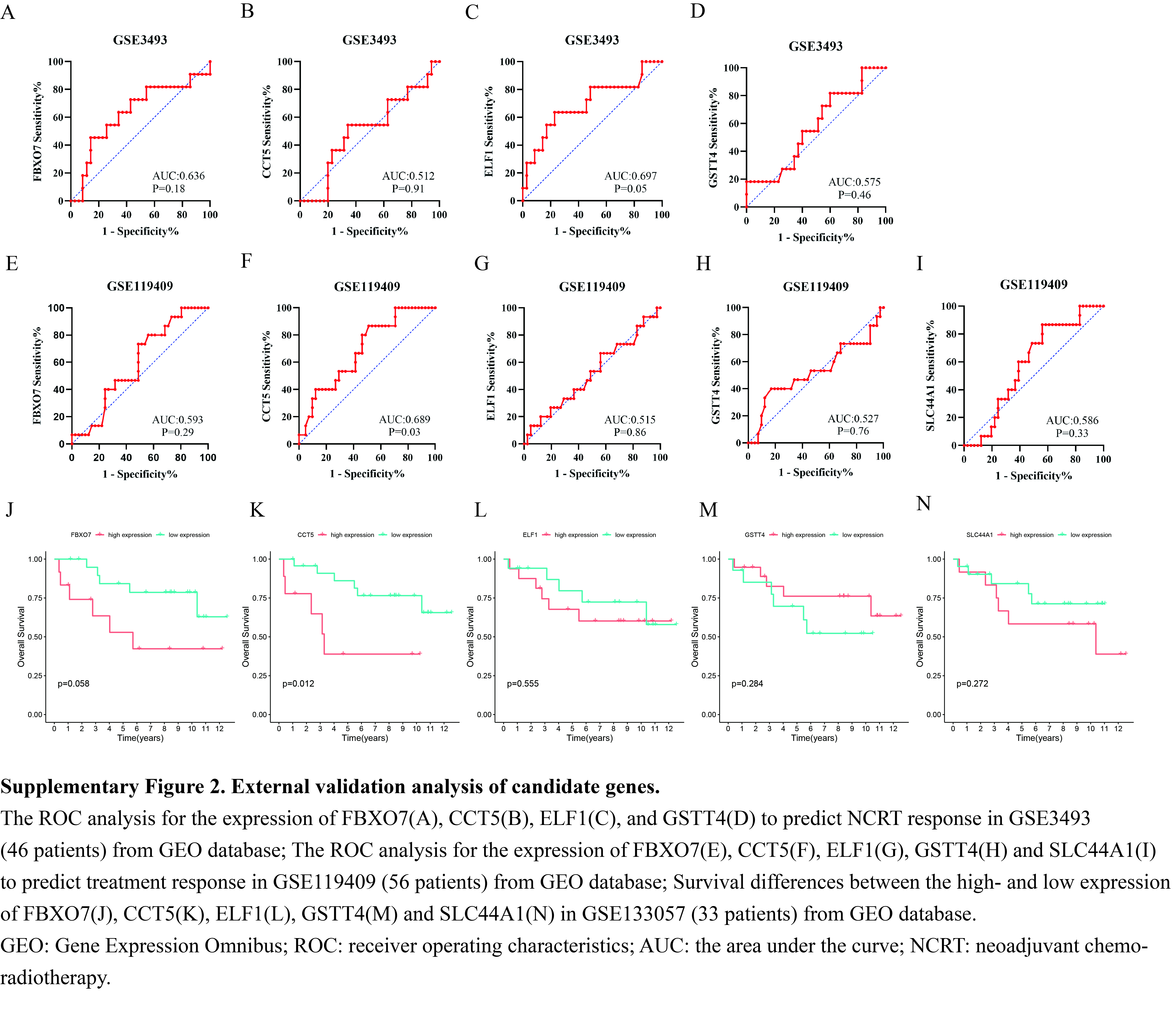

Supplement: Supplementary file 2 — Supplementary Material 2 [file 12885_2023_11354_MOESM2_ESM.tif]

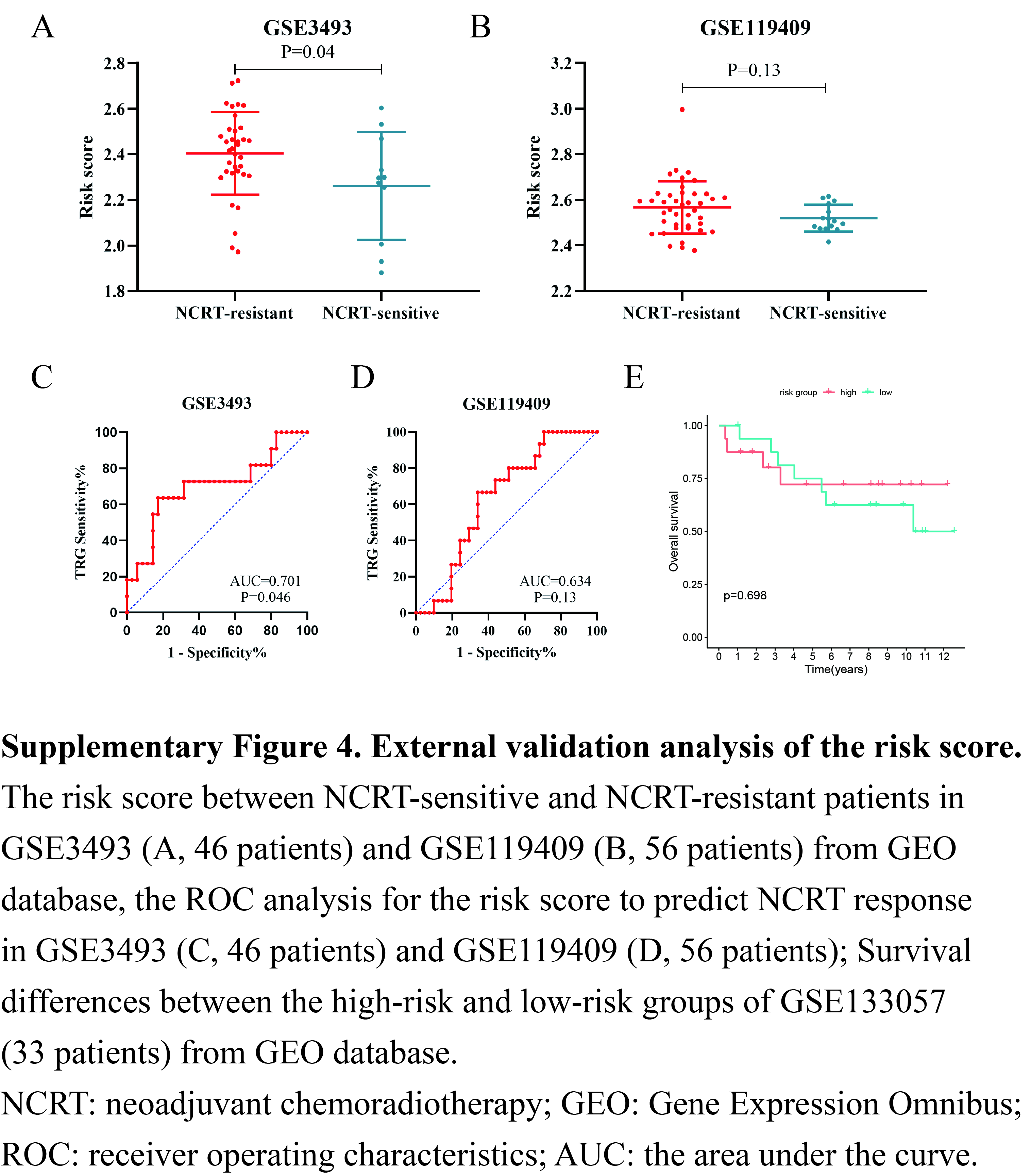

Supplement: Supplementary file 4 — Supplementary Material 4 [file 12885_2023_11354_MOESM4_ESM.tif]
