# Supplementary material for: Novel biomarkers to predict treatment response and prognosis in locally advanced rectal cancer undergoing neoadjuvant chemoradiotherapy
Source: BMC Cancer. 2023 Nov 12;23:1099. doi: 10.1186/s12885-023-11354-8 (PMC10642053; doi:10.1186/s12885-023-11354-8)
Supplement: Supplementary file 5 — Supplementary Material 5 [file 12885_2023_11354_MOESM5_ESM.docx]

Supplementary Table 1. Baseline characteristics of our cohort of 64 LARC patients.

| Characteristics | N (%） |
| --- | --- |
| Age, years |  |
| Mean ± SD | 56.6±10.1 |
| Sex |  |
| Female | 20 (31.2) |
| Male | 46 (71.9) |
| Pre-NCRT CEA (ng/ml) |  |
| ≤5 ng/ml | 36 (56.3) |
| >5 | 28 (43.8) |
| Pre-NCRT CA19-9 (U/ml) |  |
| ≤37 | 55 (85.9) |
| >37 | 9 (14.1) |
| TRG |  |
| 0 | 10 (15.6) |
| 1 | 23 (35.9) |
| 2 | 26 (40.6) |
| 3 | 5 (7.8) |
| NAR score |  |
| Median (range) | 8.4 (0-50.4) |

LARC: locally advanced rectal cancer; NCRT: neoadjuvant chemoradiotherapy; CEA: carcinoembryonic antigen; CA19-9: carbohydrate antigen 19-9; TRG: tumor regression grading; NAR score: neoadjuvant rectal-score.
