# Supplementary material for: Novel biomarkers to predict treatment response and prognosis in locally advanced rectal cancer undergoing neoadjuvant chemoradiotherapy
Source: BMC Cancer. 2023 Nov 12;23:1099. doi: 10.1186/s12885-023-11354-8 (PMC10642053; doi:10.1186/s12885-023-11354-8)
Supplement: Supplementary file 6 — Supplementary Material 6 [file 12885_2023_11354_MOESM6_ESM.docx]

Supplementary Table 2. Baseline characteristics of the validation cohort of 117 LARC patients..

| Characteristics | N (%） |
| --- | --- |
| Age, years |  |
| Mean ± SD | 56.0±11.2 |
| Sex |  |
| Female | 41 (35.0) |
| Male | 76 (65.0) |
| ASA |  |
| 1 | 85 (72.6) |
| 2 | 29 (24.8) |
| 3 | 3 (2.6) |
| Distance from the anal verge, cm | 6.6±2.7 |
| Pre-NCRT CEA (ng/ml) |  |
| ≤5 ng/ml | 70 (59.8) |
| >5 | 47 (40.2) |
| Pre-NCRT CA19-9 (U/ml) |  |
| ≤37 | 104 (88.9) |
| >37 | 13 (11.1) |
| Interval time between NCRT and surgery, weeks |  |
| Mean ± SD | 8.7±1.9 |
| ypTMN stage |  |
| ypCR | 25 (21.4) |
| ypStage I | 28 (23.9) |
| ypStage II | 38 (32.5) |
| ypStage III | 26 (22.2) |
| TRG |  |
| 0 | 25 (21.4) |
| 1 | 36 (30.8) |
| 2 | 49 (41.9) |
| 3 | 7 (6.0) |
| Pathological type |  |
| adenocarcinoma | 104 (88.9) |
| MAC or SRCC | 13 (11.1) |
| NAR score |  |
| Median (range) | 8.4 (0-50.4) |

NCRT: neoadjuvant chemoradiotherapy; ASA: American society of anesthesiologists; CEA: carcinoembryonic antigen; CA19-9: carbohydrate antigen 19-9; TNM: tumor-node-metastasis; pCR: pathological complete response; TRG: tumor regression grading; MAC: mucinous adenocarcinoma; SRCC: signet ring cell carcinoma; NAR score: neoadjuvant rectal-score.
