# Supplementary material for: Novel biomarkers to predict treatment response and prognosis in locally advanced rectal cancer undergoing neoadjuvant chemoradiotherapy
Source: BMC Cancer. 2023 Nov 12;23:1099. doi: 10.1186/s12885-023-11354-8 (PMC10642053; doi:10.1186/s12885-023-11354-8)
Supplement: Supplementary file 7 — Supplementary Material 7 [file 12885_2023_11354_MOESM7_ESM.docx]

Supplementary Table 3. Baseline characteristics of high- and low risk score groups in the validation cohort of 117 LARC patients.

| Characteristics | High risk score | Low risk score | *P* value |
| --- | --- | --- | --- |
|  | (N=57) | (N=60) |  |
| Age, years |  |  |  |
| Mean ± SD | 55.1±11.1 | 56.8±11.3 | 0.417 |
| Sex |  |  | 0.706 |
| Female | 19 | 22 |  |
| Male | 38 | 38 |  |
| ASA |  |  | 0.244 |
| 1-2 | 57 | 57 |  |
| 3 | 0 | 3 |  |
| Distance from the anal verge, cm |  |  | 0.462 |
| Mean ± SD | 6.8±2.9 | 6.4±2.4 |  |
| Pre-NCRT CEA (ng/ml) |  |  | 0.122 |
| ≤5 ng/ml | 30 | 40 |  |
| >5 | 27 | 20 |  |
| Pre-NCRT CA19-9 (U/ml) |  |  | 0.844 |
| ≤37 | 51 | 53 |  |
| >37 | 6 | 7 |  |
| Interval time between NCRT and surgery, weeks |  |  | 0.661 |
| Mean ± SD | 8.8±2.0 | 8.6±1.9 |  |
| ypTMN stage |  |  | <0.001 |
| ypCR | 4 | 21 |  |
| ypStage I | 10 | 18 |  |
| ypStage II | 24 | 14 |  |
| ypStage III | 19 | 7 |  |
| TRG |  |  | <0.001 |
| 0-1 | 17 | 44 |  |
| 2-3 | 40 | 16 |  |
| Pathological type |  |  | 0.014 |
| adenocarcinoma | 46 | 58 |  |
| MAC or SRCC | 11 | 2 |  |
| NAR score |  |  | <0.001 |
| Median (range) | 15.0 (0-50.4) | 7.5 (0-50.4) |  |

NCRT: neoadjuvant chemoradiotherapy; ASA: American society of anesthesiologists; CEA: carcinoembryonic antigen; CA19-9: carbohydrate antigen 19-9; TNM: tumor-node-metastasis; TRG: tumor regression grading; MAC: mucinous adenocarcinoma; SRCC: signet ring cell carcinoma; NAR score: neoadjuvant rectal-score.
