# Supplementary material for: Novel biomarkers to predict treatment response and prognosis in locally advanced rectal cancer undergoing neoadjuvant chemoradiotherapy
Source: BMC Cancer. 2023 Nov 12;23:1099. doi: 10.1186/s12885-023-11354-8 (PMC10642053; doi:10.1186/s12885-023-11354-8)
Supplement: Supplementary file 8 — Supplementary Material 8 [file 12885_2023_11354_MOESM8_ESM.docx]

**Supplementary Figure 1.**


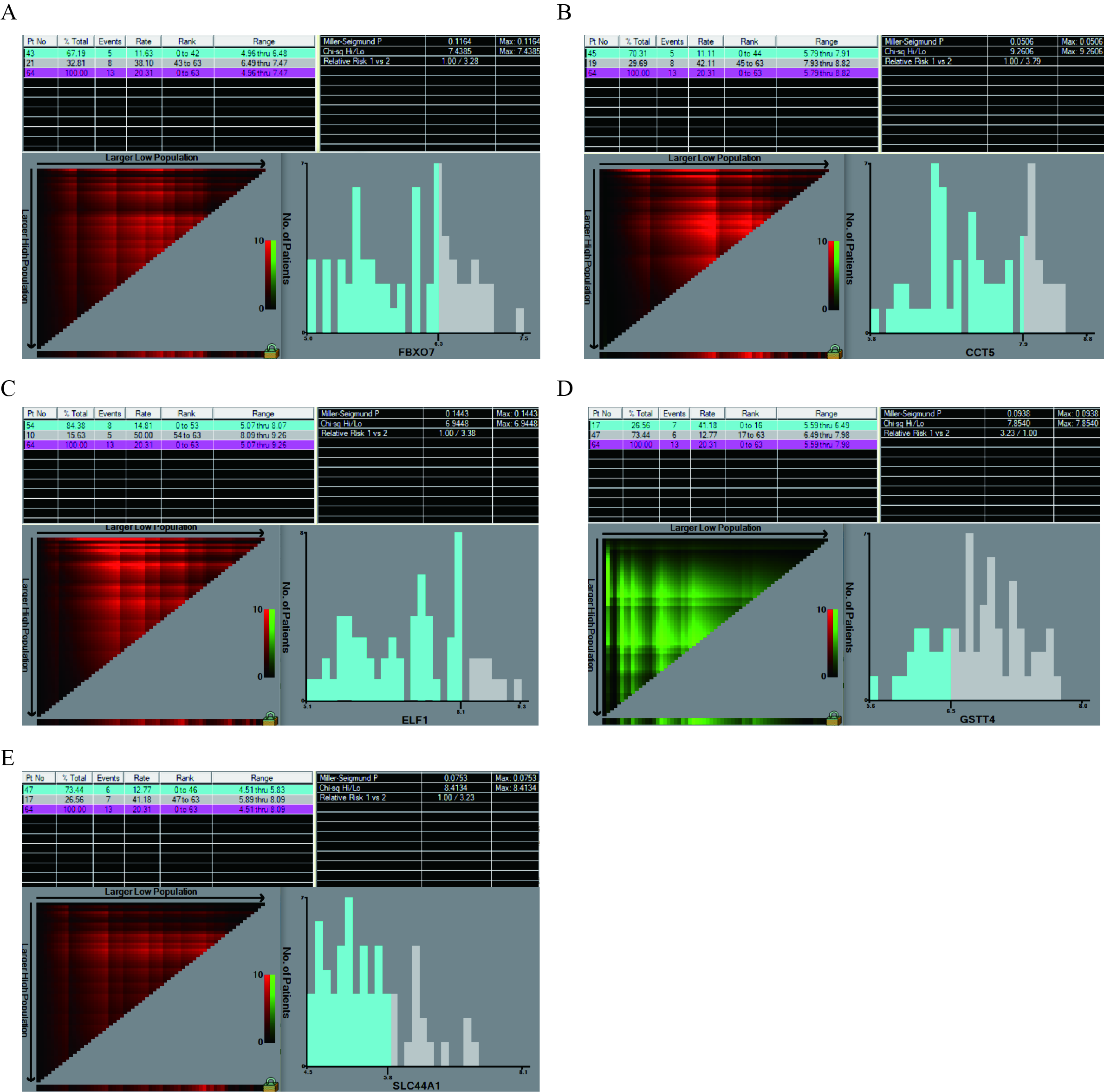


**Supplementary Figure 1.** In 64 LARC patients, the cut-off points of the expression of FBXO7(A), CCT5(B), ELF1(C), GSTT4(D), and SLC44A1(E) were identified for survival analysis by X-tile.

**Supplementary Figure 2.**


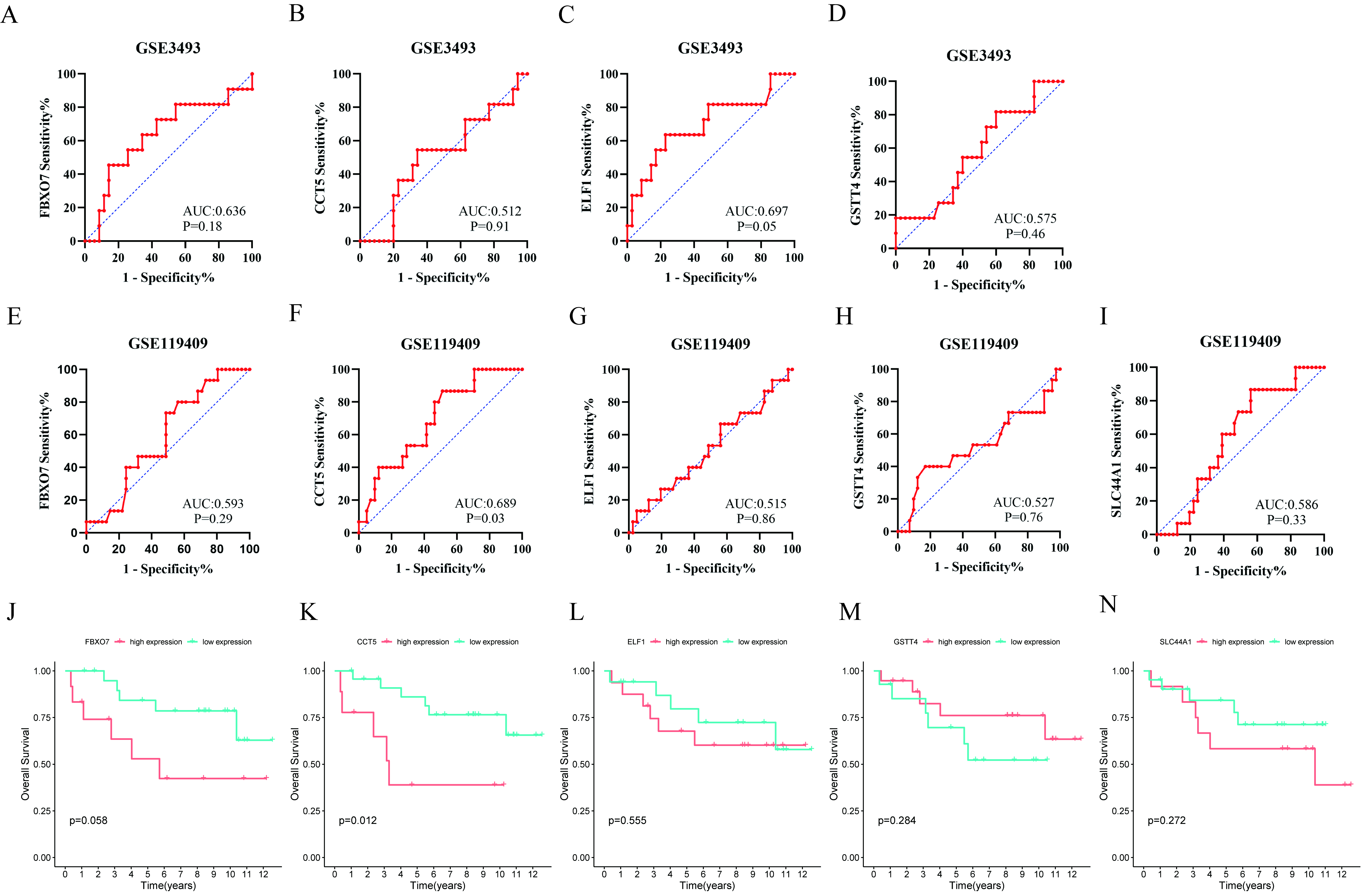


**Supplementary Figure 2. External validation analysis of candidate genes.**

The ROC analysis for the expression of FBXO7(A), CCT5(B), ELF1(C), and GSTT4(D) to predict NCRT response in GSE3493 (46 patients) from GEO database; The ROC analysis for the expression of FBXO7(E), CCT5(F), ELF1(G), GSTT4(H) and SLC44A1(I) to predict treatment response in GSE119409 (56 patients) from GEO database; Survival differences between the high- and low expression of FBXO7(J), CCT5(K), ELF1(L), GSTT4(M) and SLC44A1(N) in GSE133057 (33 patients) from GEO database.

GEO: Gene Expression Omnibus; ROC: receiver operating characteristics; AUC: the area under the curve; NCRT: neoadjuvant chemoradiotherapy.

**Supplementary Figure 3.**

**Supplementary Figure 3.** In validation cohort, immunohistochemical staining of FBXO7(A), CCT5(B), ELF1(C), GSTT4(D), and SLC44A1(E), respectively.

**Supplementary Figure 4.**


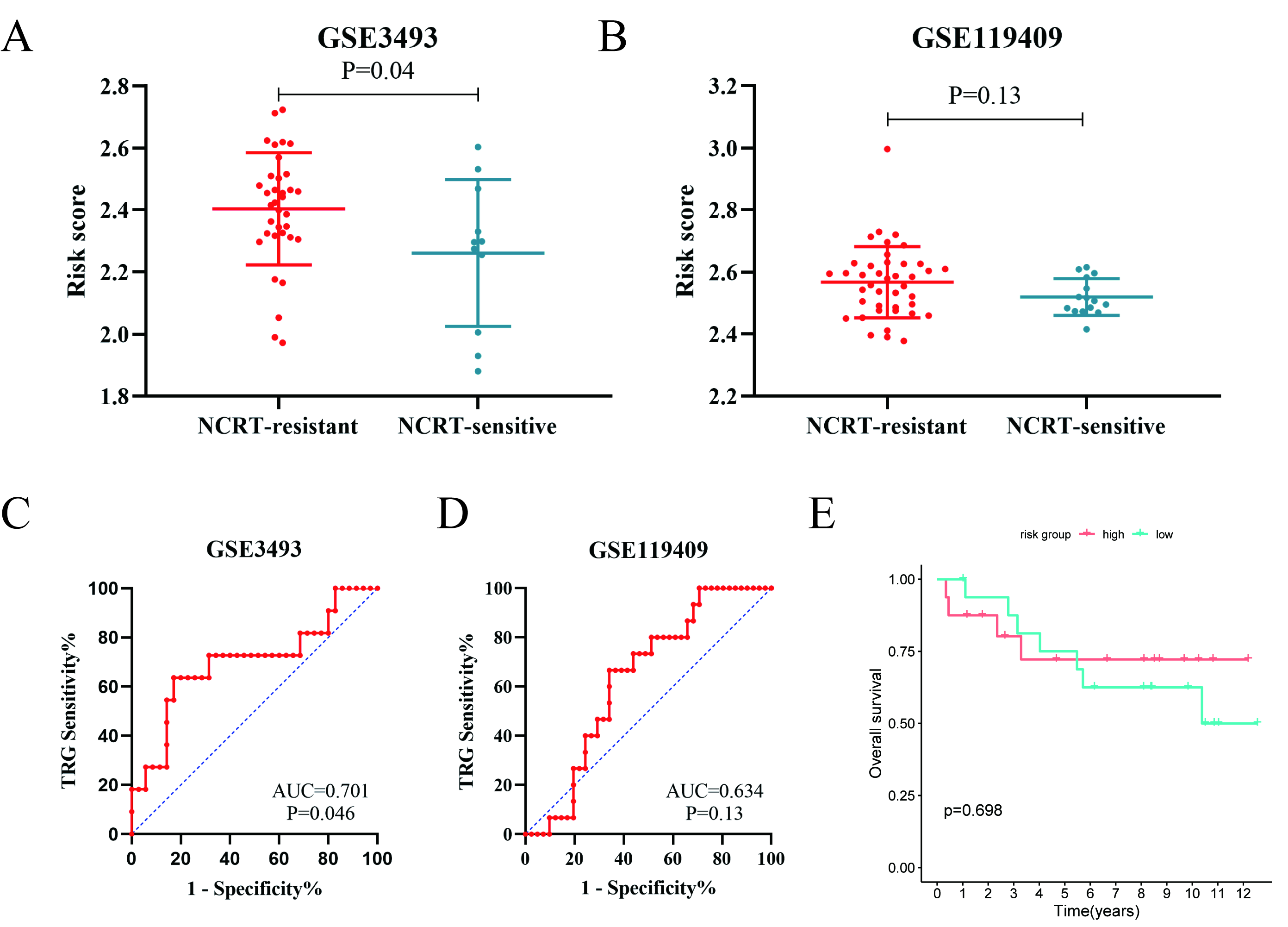


**Supplementary Figure 4. External validation analysis of the risk score.**

The risk score between NCRT-sensitive and NCRT-resistant patients in GSE3493 (A, 46 patients) and GSE119409 (B, 56 patients) from GEO database, the ROC analysis for the risk score to predict NCRT response in GSE3493 (C, 46 patients) and GSE119409 (D, 56 patients); Survival differences between the high-risk and low-risk groups of GSE133057 (33 patients) from GEO database.

NCRT: neoadjuvant chemoradiotherapy; GEO: Gene Expression Omnibus; ROC: receiver operating characteristics; AUC: the area under the curve.
